# Early-onset group B streptococcal infections in five Nordic countries with different prevention policies, 1995 to 2019

**DOI:** 10.2807/1560-7917.ES.2024.29.3.2300193

**Published:** 2024-01-18

**Authors:** Verna Björklund, Harri Saxén, Olof Hertting, Emma Louise Malchau Carlsen, Steen Hoffmann, Stellan Håkansson, Valtýr Stefánsson Thors, Ásgeir Haraldsson, Anne Karin Brigtsen, Henrik Döllner, Heikki Huhtamäki, Tytti Pokka, Terhi Susanna Ruuska, Nordic Research Network for Paediatric Infectious Diseases Study Group (NORDPID), Nordic Research Network for Paediatric Infectious Diseases Study Group (NORDPID), Anna Berggren, Terho Heikkinen, Jussi Mertsola, Ulrikka Nygaard, Ville Peltola, Marjo Renko, Jukka Uotila, Nadja Hawwa Vissing

**Affiliations:** 1 New Children's Hospital, University of Helsinki and Helsinki University Hospital, Helsinki, Finland; 2 Astrid Lindgren Children’s Hospital, Karolinska University Hospital, Stockholm, Sweden; 3 Department of Women’s and Children’s Health, Karolinska Institutet, Stockholm, Sweden; 4 Department of Intensive Care for Newborns and Infants, Copenhagen University Hospital, Rigshospitalet, Copenhagen, Denmark; 5 Neisseria and Streptococcus Reference Laboratory, Department of Bacteria, Parasites & Fungi, Statens Serum Institut, Copenhagen, Denmark; 6 Department of Clinical Science/Paediatrics, Umeå University, Umeå, Sweden; 7 Children’s Hospital Iceland, Landspitali University Hospital, Reykjavik, Iceland; 8 University of Iceland, Faculty of Medicine, Reykjavik, Iceland; 9 Department of Neonatal Intensive Care, Clinic of Paediatric and Adolescent Medicine, Oslo University Hospital, Oslo, Norway; 10 Department of Clinical and Molecular Medicine, Norwegian University of Science and Technology, Children’s Clinic, St. Olavs University Hospital, Trondheim, Norway; 11 Department of Paediatrics and Adolescent Medicine, Oulu University Hospital, Oulu, Finland; 12 Research Service Unit, Oulu University Hospital, Oulu, Finland; 13 Biocenter Oulu and Research Unit of Clinical Medicine, University of Oulu, Oulu, Finland; 14 The members of the group are listed under Collaborators

**Keywords:** Neonatal sepsis, group B streptococcus, intrapartum antibiotic prophylaxis, streptococcal screening, risk-based prophylaxis

## Abstract

**Background:**

Neonatal early-onset disease caused by group B *Streptococcus* (GBS) is a leading cause of infant morbidity. Intrapartum antibiotic prophylaxis (IAP) is effective in preventing early-onset GBS disease, but there is no agreement on the optimal strategy for identifying the pregnant women requiring this treatment, and both risk-based prophylaxis (RBP) and GBS screening-based prophylaxis (SBP) are used.

**Aim:**

The aim of this study was to evaluate the effect of SBP as a public health intervention on the epidemiology of early-onset GBS infections.

**Methods:**

In 2012, Finland started the universal SBP, while Denmark, Iceland, Norway and Sweden continued with RBP. We conducted an interrupted time series analysis taking 2012 as the intervention point to evaluate the impact of this intervention. The incidences of early- and late-onset GBS infections during Period I (1995–2011) and Period II (2012–2019) were collected from each national register, covering 6,605,564 live births.

**Results:**

In Finland, a reduction of 58% in the incidence of early-onset GBS disease, corresponding to an incidence rate ratio (IRR) of 0.42 (95% CI: 0.34–0.52), was observed after 2012. At the same time, the pooled IRR of other Nordic countries was 0.89 (95% CI: 0.80–1.0), specifically 0.89 (95% CI: 0.70–1.5) in Denmark, 0.34 (95% CI: 0.15–0.81) in Iceland, 0.72 (95% CI: 0.59–0.88) in Norway and 0.97 (95% CI: 0.85–1.1) in Sweden.

**Conclusions:**

In this ecological study of five Nordic countries, early-onset GBS infections were approximately halved following introduction of the SBP approach as compared with RBP.

**Key public health message**

**What did you want to address in this study and why?**

Early-onset group B streptococcal (GBS) disease involves sepsis, meningitis and pneumonia and is a leading cause of infant morbidity and mortality. The best prevention strategy of early-onset GBS disease is not known, but intrapartum antibiotic prophylaxis (IAP) given to mothers in labour is a widely used method. We report GBS epidemiology in five Nordic countries that use different public health strategies for identifying pregnant women eligible for IAP.

**What have we learnt from this study?**

Universal screening of pregnant women was initiated solely in Finland in 2012 and this intervention was associated with a marked reduction of 58% in the incidence of early-onset GBS infections. There was no notable change in the frequency of GBS infections in the rest of the Nordic countries using a different strategy for IAP.

**What are the implications of your findings for public health?**

Universal screening of pregnant women appeared to be an effective method to administer intrapartum antibiotic prophylaxis in preventing GBS infections in newborn infants.

## Introduction

Since the first reported neonatal cases in the 1960s [1], early-onset group B streptococcal (GBS) disease (EOD), involving sepsis, meningitis and pneumonia, has been a leading cause of infant morbidity and mortality [2]. Estimates of the incidence of EOD vary between countries, with a calculated pooled global incidence of 0.41 cases per 1,000 live births [3]. The proportion of pregnant women colonised by GBS has been in the range of 16–24% in North America and Europe [4]. Without preventive measures, 30–70% of newborns to GBS-colonised women will acquire GBS during delivery, and 1–2% of these will develop EOD [5]. Randomised clinical trials and observational studies have demonstrated the efficacy and effectiveness of intrapartum antibiotic prophylaxis (IAP) for preventing EOD, but a lack of efficacy has been noted against late-onset GBS disease [6].

There is still no international consensus on the best strategy for identifying pregnant women eligible for IAP. One option is universal screening-based prophylaxis (SBP), i.e. the screening of all pregnant women for GBS colonisation, either by culture during late pregnancy or by means of a rapid PCR-based test during labour. The other option is risk-based prophylaxis (RBP), in which mothers with known preceding risk factors are identified and treated with prophylactic antibiotics during labour [7,8]. Both approaches are currently in use. Even though there is evidence to support the use of universal GBS screening, the issue remains controversial because active universal screening may increase the use of IAP with possible negative health effects [9]. In approximately one-third of high-income countries, IAP is administered based on RBP [10], including most of the Nordic countries, the Netherlands, New Zealand and the United Kingdom, for instance. At the same time, many countries, including the United States [7,8], base their IAP programme on universal maternal screening.

The five Nordic countries, Denmark, Finland, Iceland, Norway and Sweden, share similar healthcare systems but currently have different national policies regarding the prevention of early-onset GBS disease in newborns. Finland has recommended SBP since 2012, while the other Nordic countries have continued to use RBP. In this register-based ecological study we used comprehensive national registers and conducted an interrupted time series (ITS) analysis [11] to compare the real-life effectiveness of selected public health interventions, SBP or RBP, in preventing early-onset GBS disease in newborn infants. Late-onset GBS disease cases were included in the study as an internal control measurement to reflect the overall temporal changes in the epidemiology of GBS infections.

## Methods

### Study design and supervision

This register-based ecological study of early-onset GBS sepsis incidence in five Nordic countries, Denmark, Finland, Iceland, Norway and Sweden, between 1995 and 2019 employed an interrupted time series analysis to evaluate the impact of the public health interventions involved. The Nordic Research Network for Paediatric Infectious Diseases (NORDPID), a Nordic collaboration launched in 2018 under the umbrella of the Nordic Society of Clinical Microbiology and Infectious Diseases (NSCMID) and the European Society of Paediatric Infectious Diseases (ESPID), was responsible for designing the study, which was initiated towards the end of 2019, so that the data for the individual countries were collected in the period 2019 to 2021 and the statistical analyses conducted during 2021.

### Risk-based and screening-based intrapartum antibiotic prophylaxis

The risk factors commonly used in Nordic countries for RBP purposes include premature rupture of the membranes for ≥ 18 h, a history of GBS-positive vaginal or urine cultures earlier during the pregnancy, intrapartum fever (≥ 38 °C), a previous child with early-onset GBS infection, or premature delivery at < 37 weeks. There was only minor variation in these details between the national guidelines in the Nordic countries that use RBP. For the detailed clinical indications per country, we refer to Supplementary Table S1. 

The GBS screening for SBP was performed either by conventional culture from rectovaginal swabs at 35 to 37 weeks’ gestation or by means of a rapid intrapartum vaginal/rectal PCR-based test (Xpert GBS Cepheid). In Finland, the only Nordic country using SBP since 2012, approximately half of the maternity units use intrapartum PCR-based testing, while the other half obtain cultures in late pregnancy.

In the case of both RBP and SBP, the recommended antibiotic prophylaxis was given intravenously during labour. The recommended first-line antibiotic in all these countries was penicillin-G. We did not include national recommendations for the second line antibiotics or susceptibility profiles here, because the data on intrapartum antibiotics have not been included in national registers.

### Case definition for early-onset group B streptococcal disease

For data collection purposes, a case of early-onset GBS infection was defined as any blood culture and/or cerebrospinal fluid culture denoting a positive infection in the infant within the first 6 days of life during the period 1995 to 2019 to be found in the national register for transmittable diseases or a comparable source. Late-onset cases were analogously defined as GBS infection identified at the age of 7–89 days of life. Preterm infants were included. Clinical practice regarding the diagnostics or the treatment of the suspected neonatal infections did not change in the Nordic countries during the study period.

### National registers and prevention guidelines for group B streptococcal disease in the Nordic countries

#### Denmark

Data on blood and/or cerebrospinal fluid (CSF) culture-positive cases of early-onset GBS infection were supplied by Statens Serum Institut (SSI), where individual microbiological departments send isolates on a voluntary basis. The capture rate of this database was estimated to be around 60% in a Danish study from 2005 [12]. This nationwide register is not openly available, and permission to extract the figures from the database for the present purpose was obtained locally (Danish Patient Safety Authority, reference number: 3–3013–2415/1). The estimate for GBS carriage in Denmark was retrieved from a study published in 2017 [13], and the percentage of mothers receiving IAP from one published in 2013 [14]. National guidelines for risk-based IAP were introduced in Denmark in 1997 [15].

#### Finland

We collected microbiologically verified blood and/or CSF culture-positive cases of early-onset GBS infection from the Finnish Institute for Health and Welfare (THL), which receives reports from the regional microbiological laboratories and maintains a national register of infectious diseases. This online database is open to the public [16]. The percentage of GBS carriage in Finnish pregnant women was extracted from a publication produced in 2012 [17], while the proportion of infants exposed to IAP was extracted from the same publication [17] and from the records of three university hospitals in Finland (Helsinki, Oulu and Tampere). Risk-based IAP was used exclusively in Finland during the years 1998 to 2011; thereafter, a new national guideline for universal maternal screening for GBS was released [18]. By 2014, all hospitals had adopted SBP, performed either by culture in late pregnancy or by means of PCR during labour. In Finland, two out of five university hospital districts use intrapartum PCR-based screening for GBS. Midwives perform the test during labour. The remaining three university hospitals screen GBS by obtaining bacterial culture samples at 35–37 weeks of gestation. University hospitals have independently decided whether screening is performed intrapartum or in late pregnancy. The differences in screening methods are historical: each university district has determined their own strategy and smaller hospitals usually follow the method recommended in their district.

#### Iceland

Nationwide register data for GBS disease were retrieved from the Department of Microbiology of Landspitali University Hospital in Reykjavik, which receives reports of cases positive for GBS in blood and/or CSF culture from all regional microbiology laboratories in Iceland. The register-based data were not openly available for research purposes and authorisation was obtained locally (the National Bioethics Committee, reference number: VSNb2015120015/03.03). GBS carriage rates in Iceland were extracted from a study published in 2003 [19], and the percentage of mothers receiving IAP was given as a personal information by our NORDPID collaborator, who received the unpublished data from an Icelandic study group. Iceland has had a national RBP recommendation since 1995 [20].

#### Norway

The Norwegian Surveillance System for Communicable Diseases (MSIS), a subdivision of the Norwegian Institute of Public Health, has been collecting statutory information from individual microbiological departments on blood and/or CSF culture-positive cases of early-onset GBS infections since 1986. As MSIS maintains a partially open-access database [21], the data needed for our purposes did not require official authorisation. The most recent percentage of GBS carriage in pregnant women in Norway was extracted from a study published in 2015 [22] and the estimate for the percentage of mothers receiving IAP was extracted from a national report dated 2009 [23]. The national guideline issued by the Norwegian Society of Gynaecology and Obstetrics recommending RBP was first published in 2009 [24] and updated in 2014. Furthermore, Norway has had unofficial regional guidelines with similar recommendations since 1998.

#### Sweden

Blood and/or CSF culture-positive EOD cases recorded since 2012 were retrieved from the Swedish Neonatal Quality Register (SNQ). This database presents cases observed in neonatal units, which excludes neonates already discharged from the maternity units and re-admitted to regular paediatric wards with an early-onset GBS infection. These aggregated national data are published in annual reports which are openly available on the register’s website [25]. Since the website reports the mean incidences, we retrieved the absolute annual numbers from our Swedish co-authors of the present study. For the earlier period, between 1995 to 2011, we collected the annual incidence figures from two earlier publications covering these years [26,27]. Again, these publications report the mean incidences, and we retrieved the absolute annual numbers from the authors of those publications. No data were available for 1995 to 1996 and 2002 to 2005. An estimate of GBS carriage in Sweden was obtained from a study published in 2005 [28], but the proportion of mothers receiving IAP was not available in Sweden. The Swedish guideline for RBP was published in 2008 [29].

### Statistical methods

The annual incidences of early and late-onset GBS infections per 1,000 live births with 95% confidence intervals (CI) were calculated and reported for the five Nordic countries. The data for late-onset GBS infections were retrieved from the same sources described for early-onset cases above. Sweden was excluded from the late-onset GBS infection statistics since no data on these cases were available. 

We then performed an interrupted time series (ITS) analysis, which is a quasi-experimental design that evaluates changes in longitudinal data in relation to an intervention point and can be used to evaluate the impact of public health interventions when these have been implemented at a defined point in time. This method allows detecting statistically significant changes in the incidences of early and late-onset GBS infection over the study period. The interrupted time series analysis was performed as described earlier [11].

In brief, the intervention point in the present study was the year 2012, when the IAP guideline in Finland changed from RBP to SBP. The time series of our study included an intercept time variable which consisted of the annual GBS incidences in each country in 1995 to 2019, and a period variable, i.e. Period I (1995–2011) before and Period II (2012–2019) after the intervention. Although several updates of national guidelines were released, there were no substantial changes in the maternal GBS screening instructions in other Nordic countries during the time period defined here (1995–2019).

We report the incidence rate ratios (IRRs) with 95% CI, using 2012 as the intervention time point, i.e. we compared the incidences between Period II and I in each country, including those countries with unchanged IAP policies, to evaluate the impact of the different public health policies.

## Results

### Populations

We received national register-based data covering 1,592,263 births in Denmark from 1995 to 2019, 1,423,546 births in Finland between 1995 and 2019, 108,533 births in Iceland between 1995 and 2019, 1,463,699 births in Norway between 1995 and 2019 and 2,017,523 births in Sweden from 1997 to 2001 and from 2006 to 2019, amounting altogether to 6,605,564 births (Table 1). The estimated GBS carriage and the proportion of pregnant women receiving IAP varied between the Nordic countries (Table 1).

**Table 1:** Demographic information on the five Nordic countries over the period studied here, 1995–2019

	Population in 2019	Mean annual live births 1995–2019	GBS carriage	IAP (latest estimate)	IAP strategy and key changes
Denmark	5.8 million	63,691	10–29% [14]	13% [15]	RBP 1995–2018 Selected maternal screening 2019 [15]
Finland	5.5 million	56,941	12–20% [17]	10–25% [17]	RBP 1995–2011 Universal maternal screening 2012–2019 [18]
Iceland	357,000	4,341	25% [19]	11%^a^	RBP 1995–2019 [20]
Norway	5.3 million	58,548	26% [22]	6–8% [23]	RBP 1995–2019 [24]
Sweden	10.2 million	104,501	25% [28]	NA	RBP 1995–2019 [29]

GBS: group B *Streptococcus*; IAP: intrapartum antibiotic prophylaxis; NA: not available from Sweden during the study period; RBP: risk-based prophylaxis.

^a^ Source: author VST.

### Epidemiology of early-onset group B streptococcal disease and interrupted time series analysis

**Figure 1:**
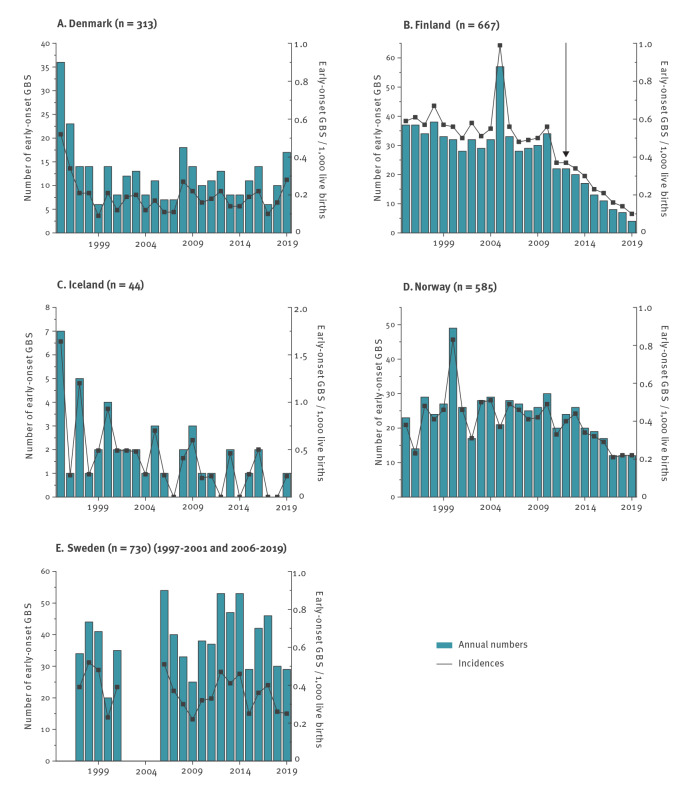
Annual numbers and incidences of early-onset group B streptococcal disease in five Nordic countries, 1995–2019 (n = 2,339)

Public health policies regarding the prevention of early-onset GBS infection in newborn infants were based on risk-based IAP during the study period in all included countries except Finland, where universal maternal screening was initiated in 2012 (Table 1). The annual incidences and absolute numbers of cases positive for GBS fluctuated in all the Nordic countries between 1995 and 2019 (Figure 1, Table 2). The proportion of women receiving IAP was highest in Finland in the era of universal GBS screening (25%) and lower in the other Nordic countries, ranging from 6% to 13% (Table 1).

GBS: Group B *Streptococcus*.

Annual numbers (columns, left y-axis) and incidences (line and symbol, right y-axis) are shown. The arrow indicates the start of universal screening in Finland.

To administer intrapartum antibiotic prophylaxis for the prevention of early-onset GBS disease Finland started universal screening for GBS in pregnant women in 2012, while the other countries continued with the risk-based approach. The y-axis scale for GBS incidence is different for Iceland because variation was larger than in the other countries. No data were available for Sweden in 1995–1996 and 2002–2005.

**Table 2:** Annual incidences and absolute case count of early-onset group B streptococcal infections in five Nordic countries, 1995–2019 (n = 2,339)

	Denmark (n = 313)		Finland (n = 667)		Iceland (n = 44)		Norway (n = 585)		Sweden (n = 730)	
	Incidence	Cases	Incidence	Cases	Incidence	Cases	Incidence	Cases	Incidence	Cases
1995	0.52	36	0.59	37	1.64	7	0.38	23	NA	NA
1996	0.34	23	0.61	37	0.23	1	0.23	14	NA	NA
1997	0.21	14	0.57	34	1.20	5	0.48	29	0.39	34
1998	0.21	14	0.67	38	0.24	1	0.41	24	0.52	44
1999	0.09	6	0.57	33	0.49	2	0.46	27	0.48	41
2000	0.21	14	0.56	32	0.93	4	0.83	49	0.23	20
2001	0.12	8	0.50	28	0.49	2	0.46	26	0.39	35
2002	0.19	12	0.58	32	0.49	2	0.31	17	NA	NA
2003	0.20	13	0.51	29	0.48	2	0.50	28	NA	NA
2004	0.12	8	0.55	32	0.24	1	0.51	29	NA	NA
2005	0.17	11	0.99	57	0.70	3	0.37	21	NA	NA
2006	0.11	7	0.56	33	0.23	1	0.49	28	0.51	54
2007	0.11	7	0.48	28	0.00	0	0.46	27	0.37	40
2008	0.27	18	0.49	29	0.41	2	0.41	25	0.30	33
2009	0.22	14	0.5	30	0.60	3	0.42	26	0.22	25
2010	0.16	10	0.56	34	0.20	1	0.49	30	0.32	38
2011	0.18	11	0.37	22	0.22	1	0.33	20	0.33	37
2012	0.22	13	0.37	22	0.00	0	0.40	24	0.47	53
2013	0.14	8	0.34	20	0.46	2	0.44	26	0.41	47
2014	0.14	8	0.30	17	0.00	0	0.34	20	0.46	53
2015	0.19	11	0.23	13	0.24	1	0.32	19	0.25	29
2016	0.22	14	0.21	11	0.50	2	0.29	17	0.36	42
2017	0.10	6	0.16	8	0.00	0	0.21	12	0.40	46
2018	0.16	10	0.14	7	0.00	0	0.22	12	0.26	30
2019	0.28	17	0.10	4	0.22	1	0.22	12	0.25	29

GBS: Group B *Streptococcus*; NA: not available.

**Figure 2:**
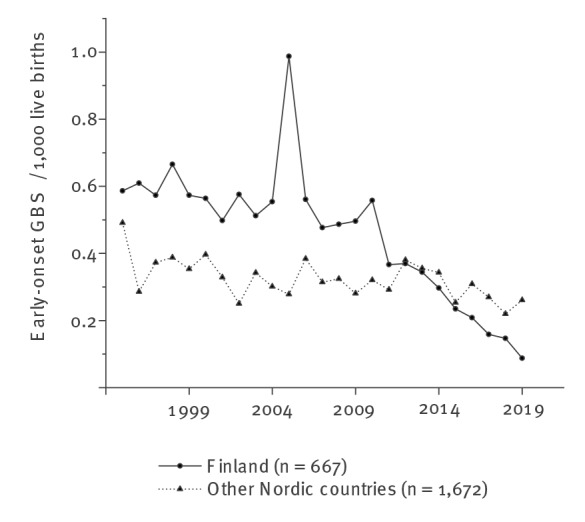
Annual incidence of early-onset group B streptococcal disease per 1,000 live births in Finland and in four other Nordic countries (Denmark, Iceland, Norway and Sweden) (n = 2,339)

The interrupted time series analysis showed a statistically significant reduction in the incidence of EOD in Finland after the introduction of universal screening for the administration of IAP in 2012 (Table 3). The IRR in Finland was 0.42 (95% CI: 0.34–0.52) when comparing Period II with Period I, i.e. a reduction by 58% (95% CI: 69–46%) was recorded. The pooled Nordic IRR in the countries not using universal maternal GBS screening, i.e. excluding Finland, was 0.89 (95% CI: 0.80–1.0), a reduction of 11% (95% CI: 21–0.0%) (Table 3), including an IRR of 0.89 (95% CI: 0.70–1.5 in Denmark, 0.34 (95% CI: 0.15–0.81) in Iceland, 0.72 (95% CI: 0.59–0.88) in Norway and 0.97 (95% CI: 0.85–1.1) in Sweden. The annual reduction in the incidence of early-onset GBS infections in Finland as compared with the other Nordic countries is also illustrated in Figure 2.

**Table 3:** Incidence rate ratios from individual countries where the incidences of Period II (2012–2019) were compared with those of Period I (1995–2011), five Nordic countries (n = 2,339)

	Early-onset GBS disease	Late-onset GBS disease
	IRR (95% CI) Period II / Period I	IRR (95% CI) Period II / Period I
Denmark	0.89 (0.70–1.5)	0.95 (0.71–1.3)
Finland	0.42 (0.34–0.52)	0.84 (0.66–1.1)
Iceland	0.34 (0.15–0.81)	1.6 (0.86–2.9)
Norway^a^	0.72 (0.59–0.88)	1.44 (1.2–1.8)
Sweden^b^	0.97 (0.85–1.1)	NA
Nordic countries excluding Finland	0.89 (0.80–1.0)^ c,d^	1.3 (1.1–1.5)^ b,e^

CI: confidence interval; GBS: group B *Streptococcus*; IRR: incidence rate ratio; NA: not available.

^a^ 1995–2011 vs 2012–2018.

^b^ 1997–2001 and 2006–2011 vs 2012–2019.

^c^ 1997–2001 and 2006–2011 vs 2012–2018.

^d ^Denmark, Sweden, Norway and Iceland.

^e ^Denmark, Norway and Iceland.

In 2012, Finland initiated universal GBS screening of all pregnant women, whereas the other Nordic countries continued to use risk-based assessment for prophylaxis.

GBS: group B *Streptococcus*.

Finland gradually started the universal screening for GBS in pregnant women in 2012, while the other Nordic countries continued with the risk-based approach to the administration of intrapartum antibiotic prophylaxis to prevent early-onset GBS disease.

### Epidemiology of late-onset group B streptococcal disease and interrupted time series analysis

The IRR for late-onset neonatal GBS sepsis in Finland was 0.84 (95% CI: 0.66–1.1) when comparing Period II with Period I. There was a 30% increase (IRR = 1.3; 95% CI: 1.1–1.5) in the incidence of late-onset GBS infections in the pooled data from those Nordic countries not using universal maternal GBS screening, i.e. excluding Finland (Table 3). The IRR was 0.95 (95% CI: 0.71–1.3) in Denmark, 1.6 (95% CI: 0.86–2.9) in Iceland and 1.44 (95% CI: 1.2–1.8) in Norway. No data were available on late-onset GBS disease cases in Sweden.

## Discussion

In this ecological register-based study in five Nordic countries, universal screening of pregnant women for GBS was associated with a reduction of 58% in the incidence of neonatal early-onset GBS infections. In the four Nordic countries where the risk-based approach was used during the whole period concerned, we observed a more modest decrease of 11% in the incidence of neonatal EOD.

Universal GBS screening of mothers is currently in routine use in the United States [7,8] and in several European countries, including Belgium, Finland, France, Germany, Italy and Spain [10]. There is an ongoing debate on the potential benefits and drawbacks of such screening interventions [9]. Criticism has primarily been focused on the possibility that screening may lead to increased use of intrapartum antibiotics, as was also recorded here. The use of intrapartum antibiotic prophylaxis during vaginal delivery was approximately twofold in Finland as compared with that in countries using a risk-based approach. Increased use of intrapartum antibiotics could lead to increased perturbation of the maternal and neonatal microbiota [30,31], which in turn has been associated with later childhood negative health outcomes [32]. Increased use of antibiotics could promote the emergence of antibiotic resistance in GBS or other neonatal pathogens [33]. Maternal anaphylaxis due to penicillin has also been regarded as a potential risk [7,8]. The present results show that the public health policy of using universal screening of mothers for GBS appeared to be associated with a more profound reduction in EOD in newborn infants than the risk-based approach, while the use of intrapartum antibiotics was approximately twofold.

The study reported here has several strong points. It was a comprehensive ecological study using high-quality national registers of the Nordic countries, and it was conducted in five countries with a population of ca 27 million inhabitants over an extensive period of 25 years. Currently, most studies addressing the effectiveness of a chosen IAP method have been retrospective cohort studies usually involving one country, one or just a few hospitals, or single national records. The healthcare systems and registers of all the Nordic countries are similar in structure, which made the comparisons reliable, and the national guidelines for the risk-based recommendations are also comparable. The case definition of early-onset GBS infection in all the Nordic registers was accurate since only cases culture-positive (blood or CSF) for GBS were included. We reported late-onset GBS disease cases to characterise possible temporal fluctuation of GBS incidence during the study period. The overall increase of late onset GBS disease in Nordic countries, excluding Finland, indicate that streptococcal infections have by and large not decreased in the society over time. Thus, these figures serve as internal controls in our study.

Our study has however some limitations. Firstly, we did not have comprehensive data on intrapartum antibiotic consumption, because such data were not included in the national registers. Secondly, it is likely that some of the temporal changes in early-onset GBS infection incidences might be explained merely by natural fluctuation in GBS incidence. Thirdly, some true GBS disease cases might have been missed due to false negative blood cultures or due to unpredictable changes in clinical practice. Also, since a validation study to confirm the comprehensiveness of the used register was available only for Denmark [34], it may render the comparison between the national data unreliable. Yet, an earlier study by Horváth-Puhó E et al. [35] reported a higher number of GBS infections in Denmark than the present study. In the study by Horváth-Puhó et al., the retrieval of cases, however, was done using ICD diagnosis codes and late-onset GBS disease cases were included [35]. Finally, both PCR-based intrapartum screening and culture-based screening in late pregnancy were used in Finland during the study. We did not compare the impact of the different GBS screening strategies. However, a previous study showed that the intrapartum PCR and late pregnancy enriched bacterial culture had comparable sensitivities in the detection of GBS in Finland [36].

## Conclusion

This register-based ecological study performed in the Nordic countries shows that the use of universal prenatal screening of pregnant women for GBS to administer intrapartum antibiotic prophylaxis is effective in preventing 58% of EOD in newborn infants. Whether the increased early antibiotic exposure of the infants is justifiable remains debatable. Further head-to-head studies are needed to elucidate the strengths of different modes of GBS prophylaxis, at least until effective maternal GBS vaccines become available. In addition, the present results emphasise the value of continuous surveillance systems and collaborative efforts for comparing the clinical impact of public health interventions.

## The members of the Nordic Research Network for Paediatric Infectious Diseases Study Group (NORDPID):

In addition to the majority of the authors (HS, OH, EM, VS, AH, AB, HD, TSR), the following members belong to the NORDPID study group: Anna Berggren (Childhood Cancer Research Unit, Department of Women´s and Children´s Health, Karolinska Institutet and Research and Development, Norrtälje Hospital, Sweden),

Terho Heikkinen (Department of Paediatrics and Adolescent Medicine, Turku University Hospital and University of Turku, Turku, Finland),

Jussi Mertsola (Department of Paediatrics and Adolescent Medicine, Turku University Hospital and University of Turku, Turku, Finland),

Ulrikka Nygaard (Department of Paediatrics and Adolescent Medicine, Copenhagen University Hospital, Rigshospitalet, Denmark),

Ville Peltola (Department of Paediatrics and Adolescent Medicine, Turku University Hospital and University of Turku, Turku, Finland),

Marjo Renko (Department of Paediatrics, University of Eastern Finland and Kuopio University Hospital, Kuopio, Finland),

Jukka Uotila (Department of Obstetrics and Gynaecology; Tampere University Hospital and Medical School of Tampere University, Tampere, Finland),

Nadja Hawwa Vissing (Department of Paediatrics and Adolescent Medicine, Copenhagen University Hospital, Rigshospitalet, Denmark).

## Supplementary Data

132000 Supplement
