# Supplementary material for: Early-onset group B streptococcal infections in five Nordic countries with different prevention policies, 1995 to 2019
Source: Euro Surveill. 2024 Jan 18;29(3):2300193. doi: 10.2807/1560-7917.ES.2024.29.3.2300193 (PMC10797658; doi:10.2807/1560-7917.ES.2024.29.3.2300193)
Supplement: Supplement [file 23-00193_BJORKLUND_Supplement.pdf]

This supplementary material is hosted by *Eurosurveillance* as supporting information alongside the article [Early-onset group b streptococcal infections in five Nordic countries with different prevention policies], on behalf of the authors, who remain responsible for the accuracy and appropriateness of the content. The same standards for ethics, copyright, attributions and permissions as for the article apply. Supplements are not edited by *Eurosurveillance,* and the journal is not responsible for the maintenance of any links or email addresses provided therein.

Supplementary table S1. Clinical indications for risk based intrapartum antibiotic prophylaxis to prevent early-onset group B streptococcal (GBS) disease in Nordic countries. Finland started universal GBS screening-based prophylaxis in 2012. Abbreviations: PROM premature rupture of membranes, GA gestational age.

	Denmark before 2019	Finland before 2012	Iceland	Norway	Sweden
Previous child with GBS	✓	✓	✓	✓	✓
GBS during current pregnancy	✓	✓	✓	✓	✓
PROM >18h	✓	✓		✓ if mother has had a positive culture earlier in the current pregnancy**	✓
GA < 37+0 weeks *	✓	✓	✓	✓ if mother has had a positive culture earlier in the current pregnancy	✓
Signs of maternal infection (mother' s temperature) *	✓	✓	✓	✓	✓

*Not IAP antibiotics (broader spectrum antibiotics)

** Added to the national guideline in 2008
